# The Role of Individual and Neighborhood Characteristics on Mental Health after a Period of Economic Crisis in the Lisbon Region (Portugal): A Multilevel Analysis

**DOI:** 10.3390/ijerph16152647

**Published:** 2019-07-24

**Authors:** Adriana Loureiro, Paula Santana, Carla Nunes, Ricardo Almendra

**Affiliations:** 1 Centre of Studies on Geography and Spatial Planning (CEGOT), Faculty of Arts and Humanities, Colégio de São Jerónimo, University of Coimbra, 3004-530 Coimbra, Portugal; 2 Centre of Studies on Geography and Spatial Planning (CEGOT) and Department of Geography and Tourism, Faculty of Arts and Humanities, Colégio de São Jerónimo, University of Coimbra, 3004-530 Coimbra, Portugal; 3 Centre for Research in Public Health and National School of Public Health, Nova University of Lisbon, Avenida Padre Cruz, 1600-560 Lisbon, Portugal

**Keywords:** mental health, mental health determinants, neighborhood environment, economic crisis, Portugal

## Abstract

Mental health is an intrinsic dimension of health influenced by individual and contextual factors. This cross-sectional study analyzes the association between the individual, neighborhood characteristics, and one’s self-assessed mental health status in the Lisbon region after an economic crisis. Via the application of multilevel regression models, the study assesses the link between one’s neighborhood environment—deprivation, low self-assessed social capital, and low self-assessed satisfaction with the area of residence—and mental health regardless of one’s individual characteristics. Constraints related to the economic crisis play an important role in the explanation of poor mental health.

## 1. Introduction

Mental disorders are one of the world’s leading causes of disability, morbidity, and mortality [1], namely premature deaths. Between 2007 and 2017, the years of life lost by mental disorders globally increased by 18.5% [2]. In 2015, depression was the major contributor to suicide deaths and the highest contributor to global disability (7.5% of all years lived with disability); anxiety disorders were ranked sixth (3.4%) [1].

The definition of mental health, according to the World Health Organization [3], goes beyond the absence of mental disorder to include the concepts of subjective well-being, perceived self-effectiveness, self-determination, autonomy, social competence, inter-generational dependency, and self-realization of one’s intellectual and emotional potential. The same organization in 2007 [4] noted that mental health is “a state of well-being in which the individual realizes his or her own abilities, can cope with the normal stresses of life, can work productively and fruitfully, and is able to make a contribution to his or her community”. Furthermore, mental health is influenced by the interaction of a set of genetic, biological, psychological, social, and contextual factors [5].

Recent scientific evidence, focused on multifactorial research, suggests that aspects related to an individual’s neighborhood environment (one’s place of residence) may influence a person’s mental health, regardless of, or beyond, his/her individual characteristics [6,7,8,9,10,11,12,13,14,15,16,17].

Neighborhood environment is understood within a holistic perspective, which integrates the complex interrelationship of multiple contextual factors that ascribe a particular value to each place [18,19,20]. Socio-environmental characteristics of the neighborhoods where people are born, grow up, live, work, and age may produce positive or negative impacts on both individual and collective health (including mental health), acting as one of its determinants [21,22,23,24,25,26]. Places, as dynamic organisms, consisting of a set of multiple and overlapping neighborhood environments and densities, can promote exposures and generate diverse vulnerabilities related to built, connective, or relational space, which negatively influence human health, particularly mental health [27].

Through this approach, several authors suggested that improving one’s living environmental conditions (contextual characteristics) is fundamental to improving the mental health of the population, by taking actions on factors such as poverty [28], deprivation [11], income [13], employment/unemployment [11,29], natural environment [30,31], built environment [12,32,33], housing [34], social networks [35], social capital [12,35], social cohesion [15], and social discrimination and security [36].

Furthermore, certain phenomena leave their mark on the environment and may lead to the transformation and adaptation of the communities, as in the cases of economic and financial crises [37,38], rapid and uncontrolled urbanization [39,40], and high unemployment rates [41,42]. These phenomena are powerful drivers of health, particularly the financial and economic crises and their impacts, as they are often associated with an increase in mental health problems [43,44], namely mental disorders [43,45], admissions to mental health facilities [43], and suicide [43,45,46]. The health impacts of an economic crisis are often uneven with certain individuals or groups of individuals being more affected than others by downward cycles in the economy. Vulnerable groups (e.g., single-parent families, individuals of lower socio-economic status, ethnic minorities, migrants, the elderly) tend to be less resilient to negative economic conditions and to report more severe mental health problems as a consequence of the effects of the economic and financial constraints on their environment [38,43,44,45].

As for Portugal, its social and economic structures were indeed impacted by the effects of the “Great Recession”, which, according to the technical definition of an economic recession (two quarters of negative growth), lasted from 2009–2011 [47]. This compelled the country to appeal for international financial support, which resulted in the program for economic adjustment applied from 2011–2014 under the directives of the International Monetary Fund, the European Central Bank, and the European Commission. This was an austerity period characterized by, on the one hand, severe cuts in public spending and, on the other hand, a tight control of public expenditure, with repercussions on: (i) unemployment: increasing from 8.8% in 2008 to 15.8% in 2012 [47]; (ii) emigration: 136,615 new emigrants registered between 2009 and 2012 [48]; and (iii) purchasing power: standards for European Union countries decreased from 81% in 2009 to 77.3% in 2013 [49].

As far as the authors know, there is still limited scientific evidence about the effect of contextual and individual factors on mental health in Portugal. Thus, the objective of this work is to analyze the association between the individual and neighborhood characteristics and the self-assessed mental health status in four municipalities of the Lisbon region (Portugal) following a period of economic crisis.

## 2. Materials and Methods

### 2.1. Study Area

**Figure 1:**
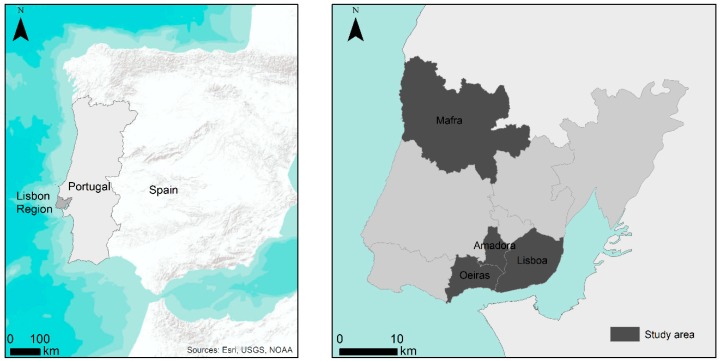
Location of study area.

The study area of this research is located within Lisbon region (according to NUT 3 (Nomenclature of Territorial Units for Statistics, Level 3) in 2011). Four municipalities were selected for their distinct geographical and socioeconomic characteristics and included consolidated urban areas (Lisbon), recent urban growth areas characterized by higher (Amadora) and lower (Oeiras) levels of deprivation and rural areas (Mafra). The four municipalities were studied at the level of civil parishes of 2011 (91 parishes) (Figure 1), the lowest local level of Portuguese administrative division. The parish scale was selected to represent and analyze the neighborhood level, in accordance with previous studies [50].

The study area had 971,674 inhabitants in 2011 (average population by parish was 8880 inhabitants, ranging between 320 and 42,306), according to the Census 2011 of Statistics Portugal [51], of which 54% were women and 46% were men. About 21% of this population was over age 65, corresponding to 61% women and 31% men. Population density varied between 75 and 19,104 inhabitants per square kilometer, with an average of 5265 inhabitants per square kilometer [48].

### 2.2. Data Collection

The primary source of information used was the questionnaire applied, between August 2014 and February of 2015, to the adult population living in the four municipalities. The survey collected information to support the assessment of the relationship between the individual and neighborhood characteristics and the self-assessed mental health status after the economic crisis (Great Recession). According to Stuckler et al. [52], the health outcomes (epidemiological data) have, normally, a latency period between two and five years; therefore, the impacts of the Great Recession on the health of the Portuguese population are still ongoing [53].

Eligibility criteria for participants in the questionnaire were: (a) being over 17 years of age at the time of the survey; (b) living in one of the selected municipalities. The Research Ethics Board at “Centro Hospitalar Lisboa Ocidental” (Hospital of Metropolitan Area of Lisbon, Portugal) provided ethics approval for this study, integrated in the research project SMAILE, Mental Health—Evaluation of the Local and Economic Determinants.

Data were collected through a representative random sample (by quota according to sex and age by municipality) of 1066 resident individuals, with a sampling error of 6% and a confidence interval of 95%. The individuals were randomly selected on the street, and data were obtained from in-person interviews conducted by trained interviewers. The response rate was 79%. Table 1 describes some characteristics of the study participants.

Individual data were collected on: (a) biological and socioeconomic characteristics, (b) behavioral characteristics and health status, (c) perception of neighborhood characteristics, (d) economic-financial constraints, and (e) mental health status.

At the ecological level, secondary data on neighborhood characteristics (material deprivation and population density) were calculated based on data provided by Statistics Portugal [51].

### 2.3. Outcome

Self-assessed mental health (MH) was measured through the mental health and vitality scales on the version validated for the Portuguese population of the Short Form 36-item Health (SF-36v2) [54]. The SF-36 is a generic health survey, which measures and assesses population health status and health-related quality of life from the individual’s point of view [55]. The mental health and vitality scales were computed following the methodology proposed by Ware et al. [56] and range from 0–100, corresponding to the situations in which the individual experiences total and no disability, respectively. The MH was converted into a dichotomous variable where scores lower or equal to 50 represent poor mental health and scores higher than 50 mean good mental health [55].

### 2.4. Neighborhood Characteristics

To analyze the neighborhood characteristics of the area of residence (parishes) that could be associated with MH, the population density (individuals per square kilometer) and a deprivation index (DI), both for 2011, were used. Population density was normalized through the z-score method. The DI was constructed with the following indicators: (a) illiteracy rate (% of people over 10 years of age unable to read or write), (b) unemployment rate (% of unemployed among the active population) and (c) substandard housing rate (% of houses without a toilet) collected in the Portuguese Statistics [51]. Other studies assessing the relationship between environmental characteristics and health also built multidimensional composite indexes to summarize neighborhood conditions [46,57,58,59]. The composite index was constructed based on the Carstairs and Morris method [60], which provides a sum of the standardized values of the indicators (using the z-score method, which applies to each indicator a weighted mean of zero and a variance of one, with the same influence on the final result). Higher index values reflect higher deprivation, and zero represents the average of all parishes in the study area. The DI was categorized into terciles (T1, T2, T3).

### 2.5. Perception of Neighborhood Characteristics

Using the individual information collected via a questionnaire on the self-assessment of one’s area of residence, two contextual scores were constructed: (a) score of satisfaction with one’s area of residence and (b) score of neighborhood’s social capital.

The first was based on the individuals’ responses to 15 questions on environmental quality, employment, and the facilities and services on offer (see Appendix A, Table A1). For each question, the response reflects qualitatively the level of satisfaction by linear gradation: from total dissatisfaction (0) to maximum satisfaction (100). The response options were converted into a quantitative scale (with intervals assuming the same gap). The score of satisfaction with the area of residence was computed through the arithmetic mean of the scores of the 15 questions.

The score of neighborhood social capital was based on Putnam’s [61] definition of social capital—linking, bonding and bridging social capital—assessing the sense of belonging and identity, family and community relational support, isolation, and trust in public institutions (see Appendix A, Table A2); Nogueira [62] used a similar approach to assess the neighborhood social capital in the Lisbon Metropolitan Area. The final scores resulting from the arithmetic mean of the score of the individual questions (calculated via the same method) were used to construct the respondents’ score of satisfaction with the area of residence.

The scores of satisfaction with the area of residence and of neighborhood social capital were categorized into a binary variable where scores lower or equal to 50 represent less satisfaction with the area of residence and lower neighborhood social capital, respectively.

### 2.6. Biological, Socioeconomic, Behavioral Characteristics, and Health Status

The association between biological and socioeconomic characteristics and MH were examined in terms of age, gender, and education level. Education level was used to represent socioeconomic characteristics. Evidence in the literature shows that educational level is one of the strongest social determinants of health, indicated as a major predictor of mortality, morbidity, health behaviors, and health literacy [63,64,65,66]. This variable was clustered into two classes: individuals having 12 or less and those with 12 years of education or more (as stipulated by Decree-Law 176/2012 dated 2 August, 12 years of compulsory education are established in Portugal). The consequences of individual behaviors on MH were assessed through the physical activity (regular physical activity: yes or no) and smoking habits (regular smoker: yes or no). The respondent’s health status was analyzed by determining whether the hypertensive status reported by the individual had been diagnosed by a doctor (yes or no).

### 2.7. Economic and Financial Constraints

The statistical relationship between economic and financial constraints and MH was analyzed by: (a) financial situation (financial situation of the household: able to save money, able to pay current expenses only, or difficulty paying expenses); (b) concerns with meeting daily expenses (more than two years ago or less than two years ago); (c) the main expenses burdening household budget (household budget mostly allocated to: health, food, education, housing, or transport); and (d) unemployed persons in the family (yes or no).

### 2.8. Statistical Analysis

To address the association between the individual and neighborhood characteristics and MH, a multilevel binary logistic regression was applied. This method allows for a 2-level structure, with individuals nested in neighborhoods; previous research already used this approach to account for the lack of spatial independence [11,13,25,67,68]. The odds ratios (ORs) of having poor MH and respective 95% confidence intervals (CIs) were calculated.

Mental health status (outcome) was initially modelled by the contextual characteristics (Model 0). Furthermore, a step-entry method was applied building on Model 0: Model 1 includes the perception of context characteristics; Model 2 adds to Model 1 by including biological, socioeconomic, and behavioral characteristics and health status information; and Model 3 builds on Model 2 by adjusting for economic and financial constraints. The step-entry sequence allowed understanding the statistical effect of adjusting for individual level risks on neighborhood level factors, along with further wider macroeconomic factors; this rationale followed the work of Chum and O’Campo [67]. During the sensitivity analysis process, the classification of the variables to be included in the models was tested (e.g., the use of tertiles), and the statistical criterion was followed; consequently, the classification that better fit the models was adopted. Furthermore, statistical interactions between variables were also tested, but the validation measures of the models did not suggest its inclusion.

Possible multicollinearity between the independent variables was assessed through a correlation matrix. The multilevel binary logistic regression was performed using SPSS Version 22 (IBM Corp., Armonk, NY, USA).

## 3. Results

Model 0 assesses the association between MH and contextual characteristics. It was found that individuals living in less deprived parishes (T1 and T2) had a significantly lower probability of having poor MH than those living in more deprived neighborhoods (OR: T1 vs. T3 = 0.71, *p* < 0.05; OR: T2 vs. T3 = 0.72, *p* < 0.05) (Table 2). Population density was not significantly associated with one’s MH status.

Model 1 builds on Model 0 by adding the score of satisfaction with area of residence and the score of neighborhood social capital as potential confounders. After adjustments, the association between mental health status and DI was no longer significant when comparing T1 with T3 (OR: 0.79, *p* > 0.05) and remained significant in T2 vs. T3 (OR: 0.68, *p* < 0.05). The two variables included as perception of neighborhood characteristics were significant predictors of MH status. Individuals with lower neighborhood social capital (OR: 2.09, *p* < 0.001) and lower satisfaction with area of residence (OR: 2.08, *p* < 0.001) had significantly higher risk of having poor MH.

Model 2 added biological, socioeconomic, behavioral characteristics, and health status—age, gender, education, physical activity, smoking behavior, and hypertension—to the previous model. The results showed that the probability of having poor MH: increased with age (OR: 1.01, *p* < 0.01); was higher for women (OR: women vs. men = 2.22, *p* < 0.001); was higher for lower educational levels, but not significantly (OR: ≤12 years of education vs. >12 years of education = 1.20, *p* > 0.05); was higher for physically-inactive individuals (OR: inactivity vs. activity = 1.50, *p* < 0.01); increased in individuals with diagnosed hypertension (OR: diagnosed vs. not diagnosed = 1.50, *p* < 0.01); and did not significantly change with smoking behavior (OR: 1.00, *p* > 0.05). The contextual characteristics and perception of context characteristics maintained the same trend presented in Model 1 (the overlapping CI of these variables can be found in the comparison Model 1 and Model 2).

Model 3 further adjusted Model 2 by including economic and financial constraints: concerns with meeting one’s daily expenses, whether one’s household budget was mostly allocated to health, the presence of unemployed individuals in the family, and one’s financial capacity. These variables were significantly associated with poor MH; they particularly highlighted the high odds ratios of having poor MH found in individuals where the majority of their household budget was allocated to health expenses (OR: health expenses vs. other expenses = 2.02, *p* < 0.001) and individuals with difficulty paying daily expenses (OR: difficulty vs. saving capacity = 1.93, *p* < 0.001). The variables included in Model 2 did not change significantly after adjusting for economic and financial constraints, with the exception of diagnosed hypertension, which was no longer significant, although its trend was holding in the same direction.

## 4. Discussion

This is the first study in Portugal exploring the association between mental health and individual and neighborhood characteristics in the Lisbon region, after a period of economic crisis. In this region, one in three individuals reported poor MH (33%).

The results show that individuals living in deprived neighborhoods reporting lower social capital and lower satisfaction with their area of residence were associated with increased odds of poor MH (*p* < 0.05) in the final adjusted model (Model 3). These significant associations between a range of neighborhood characteristics (observed: DI and self-assessed: score of neighborhood social capital and score of satisfaction with area of residence) and poor mental health at the local level are consistent with previous studies on the mental health impacts of deprivation [67,69], low social capital [8,70], and low satisfaction with area of residence [71,72]. In areas of higher deprivation, the access to collective resources (including material and social resources, such as services, housing, job opportunities, and social supports) is often threatened [57,73,74]. The health and wellbeing of residents may be harmed by the limited access to quality amenities and services [73].

Furthermore, neighborhoods with low levels of social capital promote environments with fewer supports and buffers, enhancing the negative impacts of life events on MH [40,75]. These residential environments lack the collective capacity to acquire and hold onto community resources (e.g., educational, health, housing resources) and do not promote individual behaviors intended to generate social support and safety nets [40].

The score of neighborhood social capital and the score of satisfaction with area of residence, constructed using the method presented, were applied in Portugal for the first time. Further research can validate the scales for the Portuguese population.

The dimension of urban density assessed in this study (population density) was not significantly associated with MH in any model. In the literature, some urbanization processes (linked to high urban densities) have positive effects on mental health, provided, especially, by the higher proximity to public services and commercial facilities (e.g., healthcare, green spaces, social support facilities, public transport, food stores) [72,76] and by the consequent opportunity to move around and have an active social life [76]. However, other authors linked high densities with a higher risk of MH problems [77,78,79,80]. Denser urban areas can be also related to higher risk of mental health problems mainly due to built environment characteristics, influencing the sense of belonging and identity and family, and community relational support and networks [77,78,81].

Model 2 indicated that women and physically-inactive individuals had increased odds of poor MH (*p* < 0.05). Previous studies found the same pattern [82,83]. Women suffer more from mood disorders, anxiety, and phobias [83] and use more MH services [84], when compared with men. However, women are more likely to be treated for an MH problem because they are more likely to report symptoms and display signs of common mental illnesses [85]. Physical activity has been shown to intermediate the relationship between neighborhood characteristics and MH through the levels of security (crime rates) [86] or traffic and noise [87]. Scientific evidence showed that the relationship between neighborhood characteristics and MH is frequently indirect [76,88]. People are less likely to adopt healthier behaviors if the environment where they live fails to support their choices. A healthy neighborhood environment can promote opportunities for healthy behaviors and lifestyles, supporting social and environment interactions, which in turn may result in better mental health [88]. For instance, individuals may feel restrained from getting out (e.g., meet others, walking around, do exercise) if the immediate residential environment does not provide conditions to make them feel safe or to access green/blue and recreational areas [88,89].

This is an observational transversal study, and therefore, the existence of statistical associations between the characteristics of neighborhood environment and mental health should not be interpreted in terms of causality [89]. Lee et al. [90] mentioned that ecological or cross-sectional studies have limitations related to multiple confounding factors and long time lags between exposure to neighborhood elements and the manifestation of effects. Limitations linked to the use of cross-sectional data that may have impacts on our results are the inability to address the direction of the statistical association and to assess the influences of an individual’s earlier life. Another information bias is related to the usage of self-reported information for both result and exposure variables, which can be influenced by memory bias or by social desirability [91].

Multiple dimensions associated with mental health were assessed; however, the area under ROC curve suggested that other important factors in this relationship were not included, namely due to constraints related to the availability of data at the neighborhood level, which are still to be addressed in future studies.

This study’s results consistently showed that economic and financial constraints were significantly associated with poor MH, when adjusted for the effects of neighborhood and individual factors, as shown in Model 3. The results, measured after the economic downturn, may have been influenced by this crisis context, considering the higher risks of poor MH related to: (i) the allocation of health expenses as the main expense of one’s household budget; (ii) the households’ financial situation of difficulty in paying one’s expenses; (iii) the highest concern with meeting one’s daily expenses; and (iv) the presence of unemployed individuals in the family.

Although a direct comparison with previous studies is limited, as our study did not measure the impact of the economic crisis, the results presented here are still comparable to a certain extent [92]. The international literature indicates that financial difficulties (e.g., lack of saving capacity or difficulty in paying one’s expenses and debts), resulting from an economic and financial crisis given the increase of unemployment and/or cuts in public budgets, are associated with poor MH [93,94,95,96]. These mediating and moderating mechanisms related to economic shocks and fiscal austerity measures tend to increase the risk of mental health problems [97], contributing to higher socio-material vulnerability and inequity [98,99].

In Portugal, several austerity measures focusing on reducing public expenditure were implemented via the Economic Adjustment Programme, which had negative consequences on population health and health inequalities [100,101,102,103]. The budget cuts in the health and social support sectors influenced the access to services, namely healthcare services, with a decrease in patient transportation support and an increase of user charges [47,104,105,106]. Moreover, several mental health consequences were observed during and after the Great Recession, as in the increase of: (i) the prevalence of mental disease [107], (ii) suicide [46,108], (iii) the use of the psychiatric services (inpatient units and outpatient clinics) [109], (iv) the demand for mental health emergency departments [110], (v) the under-treatment and discontinuity of care for individuals with mental health problems [111], and (vi) the use of psychotropic medications (anti-depressants and anxiolytics) [107].

The results obtained are important contributions to the current body of literature on the association between individual and neighborhood characteristics and mental health in the aftermath of the Great Recession. The promotion of mental health should include the integration of mental health into all policies and the multisectoral cooperation (e.g., urban planning and public health fields) [112] and should be present in several strategic documents on mental health (Worldwide: Mental Health Action Plan 2013–2020 [113]; Europe: European Framework for Action on Mental Health and Wellbeing 2016 [114]; and Portugal: Portuguese National Program For Mental Health 2017 [115]). The development of further research on strategic assessment of the impacts of environmental factors and neighborhood elements should be encouraged to better understand the mechanisms and the pathways of mental health.

## 5. Conclusions

The study provided evidence of the multidimensionality of the phenomenon of mental health in the Lisbon region after a context of crisis: the neighborhood characteristics played an important role regardless of one’s individual characteristics. The findings obtained are important contributions that have increased our understanding of the impacts of neighborhood environment on MH, considering the vulnerability and lack of opportunities that some urban characteristics, such as deprivation or social capital, can generate.

Thus, the improvement of MH is a shared multilevel challenge that can be addressed with an interdisciplinary approach, considering the complexity of the relationship between individuals and environments. The scientific knowledge resulting from this work contributes to better informing decision makers (e.g., professionals of urban planning and public health), and consequently, supporting intersectoral policies and interventions that prevent mental disorders and promote mental health, especially during periods of crisis.

## Figures and Tables

**Table 1:** Description of the study participants.

Variables	Categories	*N*	%
**Gender**	Female	573	53.8%
	Male	493	46.2%
**Age group**	18–29	172	16.1%
	30–44	319	29.9%
	45–59	246	23.1%
	60–74	202	19.0%
	≥75	127	11.9%
**Mental health**	Good mental health (score > 50)	715	67.1%
	Poor mental health (score ≤ 50)	351	32.9%
**Educational level**	≤12 years	770	72.2%
	>12 years	296	27.8%
**Physical activity**	Yes	525	49.2%
	No	541	50.8%
**Hypertension**	Yes	300	28.2%
	No	756	70.9%
	Missing data	10	0.9%
**Smoking habits**	Yes	275	25.8%
	No	775	72.7%
	Missing	16	1.5%
**Financial situation of the household**	Able to save money	533	50.0%
	Able to pay current expenses only	377	35.4%
	Difficulty paying current expenses	146	13.7%
	Missing	10	0.9%
**Concerns with meeting daily expenses**	More than two years ago	676	63.4%
	Less than two years ago	390	36.6%
**Main expenses burdening household budget**	Household budget mostly allocated to health	194	18.2%
	Household budget mostly allocated to other expenses (e.g., food, education, housing, transport)	863	81.0%
	Missing	9	0.8%
**Unemployed person in** **the family**	Yes	430	40.4%
	No	624	58.5%
	Missing	12	1.1%

**Table 2:** Multilevel binary logistic regressions of mental health (outcome). T, tercile.

Mental Health: Odds Ratio (95% CI) Total (*n* = 1.066)		Model 0	Model 1	Model 2	Model 3
**Neighborhood characteristics**	**Deprivation** T1 (low) vs. T3 ^a^ (high)	0.71 * (0.51–0.99)	0.79 (0.56–1.01)	0.81 (0.55–1.20)	0.80 (0.53–1.19)
	T2 vs. T3 ^a^	0.72 * (0.52–0.98)	0.68 * (0.49–0.94)	0.65 * (0.46–0.93)	0.65 * (0.45–0.95)
	**Population density**	1.01 (0.88–1.16)	0.95 (0.83–1.10)	0.95 (0.81–1.11)	0.91 (0.77–1.07)
**Perception of neighborhood characteristics**	**Score of neighborhood social capital** Low vs. high ^a^	-	2.09 *** (1.41–3.10)	2.21 *** (1.46–3.34)	2.07 ** (1.33–3.23)
	**Score of satisfaction with area of residence** Low vs. high ^a^	-	2.08 *** (1.58–2.72)	1.85 *** (1.39–.46)	1.76 *** (1.31–2.40)
**Biological, socioeconomic, behavioral, and characteristics and health status**	**Age**	-	-	1.01 (1.00–1.02)	1.01 (1.00–1.02)
	**Sex** Female vs. male ^a^	-	-	2.29 *** (1.72–3.06)	2.19 *** (1.61–2.97)
	**Education** ≤12 vs. >12	-	-	1.10 (0.77–1.59)	0.900.61–1.33)
	**Physical activity** Inactivity vs. activity ^a^	-	-	1.50 ** (1.13–1.99)	1.41 * (1.05–1.91)
	**Hypertension** Yes vs. no ^a^	-	-	1.50 * (1.08–2.10)	1.40 (0.98–1.99)
	**Smoking habits** Yes vs. no ^a^	-	-	1.00 (0.71–1.41)	0.88 (0.61–1.26)
**Economic and financial constraints**	**Household financial situation**				
	Difficulty paying expenses vs. Able to save money ^a^	-	-	-	1.93 ** (1.22–3.05)
	Able to pay current expenses only vs. able to save money ^a^	-	-	-	1.75 ** (1.25–2.44)
	**Concerns with meeting daily expenses** More than two years ago vs. Less than two years ago ^a^	-	-	-	1.59 ** (1.14–2.20)
	**Main expense of the** **household budget** Health expenses vs. other categories ^a^	-	-	-	2.02 *** (1.36–2.99)
	**Unemployed person in** **the family** Yes vs. no ^a^	-	-	-	1.50 ** (1.11–2.03)
**Corrected Akaike (AICc)**		4482.8	4532.5	4585.0	4536.3
**Precision**		66.5	67.5	69.3	72.3
**Area under ROC curve**		0.50	0.54	0.59	0.64

* *p* < 0.05; ** *p* < 0.01; *** *p* < 0.001; ^a^ OR reference class.

## Appendix A

**Table A1:** Questions and respective response scales used to construct the score of satisfaction with area of residence.

Questions (Are you Satisfied with:)	Answer Options (Score Attributed to Each Option)					
**1. Local Commerce?**	Very satisfied (100)	Satisfied (66)		Not very satisfied (33)		Not satisfied (0)
**2. Outdoor leisure spaces?**	Very satisfied (100)	Satisfied (66)		Not very satisfied (33)		Not satisfied (0)
**3. Health services and facilities?**	Very satisfied (100)	Satisfied (66)		Not very satisfied (33)		Not satisfied (0)
**4. Education services and facilities?**	Very satisfied (100)	Satisfied (66)		Not very satisfied (33)		Not satisfied (0)
**5. Cultural services and facilities?**	Very satisfied (100)	Satisfied (66)		Not very satisfied (33)		Not satisfied (0)
**6. Sports services and facilities?**	Very satisfied (100)	Satisfied (66)		Not very satisfied (33)		Not satisfied (0)
**7. Public Transport?**	Very satisfied (100)	Satisfied (66)		Not very satisfied (33)		Not satisfied (0)
**8. Parking?**	Very satisfied (100)	Satisfied (66)		Not very satisfied (33)		Not satisfied (0)
**9. Safety?**	Very satisfied (100)	Satisfied (66)		Not very satisfied (33)		Not satisfied (0)
**10. Cleaning (e.g., garbage collection, urban cleaning)?**	Very satisfied (100)	Satisfied (66)		Not very satisfied (33)		Not satisfied (0)
**11. Job offers?**	Very satisfied (100)	Satisfied (66)		Not very satisfied (33)		Not satisfied (0)
**12. Community Spaces (e.g., associations, recreation centers, clubs)?**	Very satisfied (100)	Satisfied (66)		Not very satisfied (33)		Not satisfied (0)
**13. Indoor noise levels (at home)?**	Very good (100)	Good (75)	Acceptable (50)		Bad (25)	Very bad (0)
**14. Outdoor noise levels?**	Very good (100)	Good (75)	Acceptable (50)		Bad (25)	Very bad (0)
**15. Outdoor air quality?**	Very good (100)	Good (75)	Acceptable (50)		Bad (25)	Very bad (0)

Note: The satisfaction with residence score results from the arithmetic mean of the score of the questions. A minimum of 7 answers were required.

**Table A2:** Questions and respective response scales used to construct the score of neighborhood social capital.

Questions	Answer Options (Score Attributed to Each Option)							
**1. Do you live alone?**	Yes (100)				No (0)			
**2. Do you like living in your parish?**	Like very much (100)	Like (75)		Neither like nor dislike (50)		Do not like (25)		Dislike (0)
**3. How do you describe your relationship with your neighbors in the last year?**	Much better or better than usual (100)		Worse than usual (66)		Much worse than usual (33)		No relation with neighbors (0)	
**4. When in need of financial support who do you ask?**	Neighbors (100)	Family and/or friends (75)		Bank (50)		Social solidarity institutions (25)		Nobody (0)
**5. When in need of emotional support who do you ask?**	Neighbors (100)	Family and/or friends (75)		Health professionals (50)		Social solidarity institutions (25)		Nobody (0)
**6. Did you vote in the last municipal elections?**	Yes (100)				No (0)			

Note: Satisfaction with residence score results from the arithmetic mean of the score of the questions. A minimum of 4 answers were required.
